# Cutaneous Metastasis of Cholangiocarcinoma: Report of Two Cases

**Published:** 2013-11-01

**Authors:** B. Geramizadeh, R. Giti, S. A. Malekhosseini

**Affiliations:** 1 *Department of Pathology*; 2 *Transplant Research Center*; 3 *Department of Surgery, Transplant Ward, Shiraz University of Medical Sciences, Shiraz, Iran*

**Keywords:** Cholangiocarcinoma, Skin, Metastasis

## Abstract

Cutaneous metastasis of cholangiocarcinoma is extremely rare. It can be seen at distant locations or at the site of biliary drainage. To the best of our knowledge less than 30 cases have so far been reported in the English literature. This event should be considered in every skin lesion in a patient with cholangiocarcinoma and be treated promptly by resection and chemotherapy to increase the patient’s survival. Herein, we report our experience with two patients with cholangiocarcinoma and cutaneous metastasis at the site of biliary drainage.

## INTRODUCTION

Cutaneous metastases of internal malignancies are rare and the reported incidence is about 0.7%–9% [1]. Cholangiocarcinoma is an uncommon malignancy comprising less than 2% of all cancers [2]. With an incidence of almost 0.0002%, cutaneous metastasis of cholangiocarcinoma is very rare [1]. To the best of our knowledge, less than 30 cases of metastatic cholangiocarcinoma to the skin and subcutaneous tissue have so far been reported in the English literature [3].

Some of the reported cases have been distant metastasis, especially to the scalp [3], however, in around 20 cases cutaneous metastasis was occurred via percutaneous drainage and catheterization [3]. Herein, we report on two cases of cutaneous metastasis of cholangiocarcinoma at the site of biliary drainage.

## CASE PRESENTATION

Case 1

A 38-year-old man with a 2-year history of primary sclerosing cholangitis, one year post liver transplantation presented with a subcutaneous mass at the site of biliary drainage. He had a biliary drain for about three months before the transplantation. His laboratory results at the time of presentation with subcutaneous mass included a white blood cell count (WBC) of 10,400/µL, hemoglobin level of 9.6 g/dL, alanine aminotrasferase (ALT) of 43 IU/L, aspartate aminotransferase (AST) of 40 IU, and a total bilirubin level of 1.5 mg/dL.

**Figure 1:**
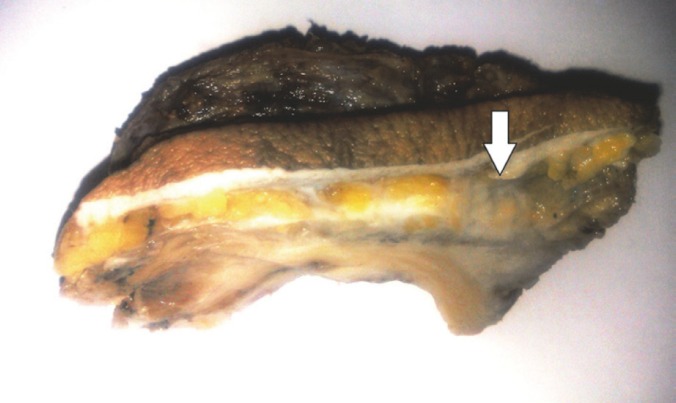
Gross view of the subcutaneous mass (arrow).

The patient was found to have cholangiocarcinoma after pathologic examination of the explanted liver, however, he did not receive any medications other than immunosuppressives for the liver transplantation. The large subcutaneous mass (4 cm in its greatest diameter) was resected (Fig 1); pathologic examination showed metastatic adenocarcinoma consistent with the previous morphology of intrahepatic cholangiocarcinoma. Now, after three months, he is still alive.

Case 2

This 41-year-old man had a 2-month history of cholangiocarcinoma. The diagnosis was made based on a liver biopsy and confirmed by immunohistochemical staining. He presented with percutaneous biliary drainage for one month and developed an abscess-like lesion at the site of previous drain in the right upper quadrant with bloody discharge.

His laboratory examination at the time of presentation was a WBC of 12,400/µL, hemoglobin level of 8 g/dL, ALT of 226 IU/L, AST of 410 IU/L, and a total bilirubin level of 21.5 (direct: 13.8) mg/dL.

**Figure 2:**
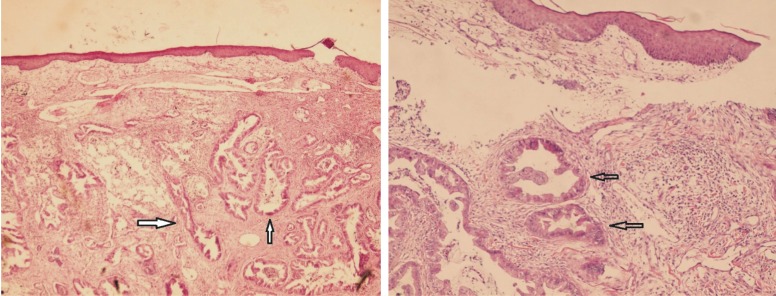
Sections from the skin show malignant glands (arrow) with mucin in the subcutaneous area. a) H&E 100×, b) H&E 400×

The patient received antibiotics without complete response. Gradually, the abscess became firm and a mass-like lesion was formed, measuring about 4 cm in diameter. The mass was excised the pathological examination of which revealed a mucin-producing adenocarcinoma, consistent with the previous morphology of cholangiocarcinoma (Fig 2). At the time of diagnosis, the patient was under chemotherapy (in another center), but unfortunately he died after less than a month after the diagnosis of cutaneous metastasis was made.

## DISCUSSION

Since 1973, percutaneous transhepatic biliary decompression has been used as a diagnostic and therapeutic means in patients with obstructive jaundice [4]. This procedure can improve hepatic function before operation and reduce post-operative morbidity and mortality in patients with jaundice and both benign and malignant diseases. It is also recommended for palliative treatment in patients with unresectable tumors [5]. However, the risks and benefits of using such drains in malignant cases should be considered [6]. Port-site metastasis is an unusual, but possible, complication of this procedure [5]. In one report on 133 cases with cholangiocarcinoma, the incidence of catheter tract implantation metastasis was almost 3% [7]. Therefore, some of the previous reports believe that, this procedure should be avoided when curative surgery is planned [8].

The period between placement of the drain and diagnosis of the cutaneous metastasis in cholangiocarcinoma is between 3 and 30 months [3]. During this period the patient should be under strict follow-up for the possibility of catheter tract subcutaneous metastasis [7].

Most of the reports on cutaneous metastasis in cholangiocarcinoma reveal that the metastasis is usually presented as a sudden-onset rapid-growing firm painless nodules with different clinical pictures including abscess, infection, panniculitis and scar tissue [6].

Some reports showed that catheter tract subcutaneous metastasis is mostly occur in well-differentiated cholangiocarcinoma. In the 20 previously reported cases, only one had poorly differentiated neoplasm [7]. Both of our cases had well-differentiated tumors.

Although cutaneous metastasis is a sign of advanced disease with 6–18 months survival [9], early diagnosis of the metastasis, as expected, can increase the patient’s survival and decrease the mortality by prompt resection of the mass and chemotherapy [6].

Case 2 was a middle-aged man with 2-month history of cholangiocarcinoma who developed an abscess-like lesion at the site of biliary drain. He died after less than one month of the diagnosis of the metastasis. He had been treated with a diagnosis of abscess for a while. However,Case 1 was promptly diagnosed; the subcutaneous mass was resected in less than a week, and he is still alive.

The problem of how to prevent catheter tract implantation metastasis in cholangiocarcinoma is an important issue yet to be solved. One proposed procedure is to excise the whole catheter tract between the surface of the skin and the punctured intrahepatic bile ducts, which can be difficult [10].

In conclusion, cutaneous metastasis of cholangiocarcinoma at the site of biliary drainage is though rare, should be considered as a poor prognostic sign of the disease and be treated promptly.
